# Effect of brief daily resistance training on rapid force development in painful neck and shoulder muscles: randomized controlled trial

**DOI:** 10.1111/cpf.12041

**Published:** 2013-06-11

**Authors:** Kenneth Jay, mc schraefel, Christoffer H Andersen, Frederik S Ebbesen, David H Christiansen, Jørgen Skotte, Mette K Zebis, Lars L Andersen

**Affiliations:** 1 National Research Centre for the Working EnvironmentCopenhagen Ø, Denmark; 2 Electronics and Computer Science Faculty of Physical and Applied Sciences, University of SouthamptonSouthampton, UK; 3 Department of Occupational Medicine Danish Ramazzini Centre, Regional Hospital HerningHerning, Denmark; 4 Institute of Sports Science and Clinical Biomechanics, University of Southern DenmarkOdense, Denmark; 5 Gait Analysis Laboratory, Hvidovre HospitalHvidovre, Denmark

**Keywords:** fear, gate control, neck and shoulder pain, rehabilitation, resistance training, threat

## Abstract

*Objective*: To determine the effect of small daily amounts of progressive resistance training on rapid force development of painful neck/shoulder muscles.

*Methods*: 198 generally healthy adults with frequent neck/shoulder muscle pain (mean: age 43·1 years, computer use 93% of work time, 88% women, duration of pain 186 day during the previous year) were randomly allocated to 2- or 12 min of daily progressive resistance training with elastic tubing or to a control group receiving weekly information on general health. A blinded assessor took measures at baseline and at 10-week follow-up; participants performed maximal voluntary contractions at a static 90-degree shoulder joint angle. Rapid force development was determined as the rate of torque development and maximal muscle strength was determined as the peak torque.

*Results*: Compared with the control group, rate of torque development increased 31·0 Nm s^−1^ [95% confidence interval: (1·33–11·80)] in the 2-min group and 33·2 Nm s^−1^ (1·66–12·33) in the 12-min group from baseline to 10-week follow-up, corresponding to an increase of 16·0% and 18·2% for the two groups, respectively. The increase was significantly different compared to controls (*P*<0·05) for both training groups. Maximal muscle strength increased only ∼5–6% [mean and 95% confidence interval for 2- and 12-min groups to control, respectively: 2·5 Nm (0·05–0·73) and 2·2 Nm (0·01–0·70)]. No significant differences between the 2- and 12-min groups were evident. A weak but significant relationship existed between changes in rapid force development and pain (*r* = 0·27, *P*<0·01), but not between changes in maximal muscle strength and pain.

*Conclusion*: Small daily amounts of progressive resistance training in adults with frequent neck/shoulder pain increases rapid force development and, to a less extent, maximal force capacity.

## Introduction

Work related musculoskeletal pain is a common problem and represents a major socioeconomic burden in the developed world. Several studies have found musculoskeletal pain to be frequent in the general population (Ferrari & Russell, 2003; Ihlebaek *et al*., 2007). For instance, in representative population of more than 29 000 people the three-month prevalence of spinal pain was 31% (Strine & Hootman, 2007). Musculoskeletal pain is typically associated with decreased muscle strength (Brox *et al*., 1997; Itoi *et al*., 1997; Sjøgaard *et al*., 2006). However, in daily life many types of events are characterized by a limited time to develop force – for instance during postural coordination and control strategies. Thus, in clinical rehabilitation settings and research the ability to rapidly develop force – that is, the rate of force development (RFD) – seems far more relevant as an outcome measure than maximal force capacity.

The literature suggests a multitude of physiological factors influencing rapid force development; from neural drive (Cutsem *et al*., 1998; Andersen & Aagaard, 2006; Holtermann *et al*., 2007) to maximal muscle strength (Andersen & Aagaard, 2006). In patients with chronic musculoskeletal pain however, sudden movements where force develops rapidly may inflict a threat response (Al-Obaidi *et al*., 2000; Carleton *et al*., 2006) and thereby limit rapid force development by pain inhibition of motor outflow (Steingrímsdóttir *et al*., 2004; Andersen *et al*., 2008a). In a previous study, markedly reduced muscle activation ability during the initial phase of a maximal voluntary contraction, measured as the rate of torque development (RTD) and rate of EMG rise, was found in women with trapezius myalgia when comparing them to healthy age-, gender- and job-matched controls (Andersen *et al*., 2008a). In that study, maximal force capacity and rapid force development were reduced by 18% and 54%, respectively (Andersen *et al*., 2008a). Thus in painful conditions, it seems that the ability to rapidly generate force is markedly impaired (Andersen *et al*., 2008a), which again may affect working capacity and daily life activities negatively. RTD may therefore be a more sensitive measure compared to maximal muscle strength, and more responsive to resistance training rehabilitation strategies.

Studies have shown an increase in RFD in healthy young (Aagaard *et al*., 2002) and elderly individuals (Suetta *et al*., 2004) following resistance training, which is congruent with typical physiological adaptations observed on rapid force- and maximal force capacity (Andersen *et al*., 2009). Moreover, significant pain relief in response to specific strength training in women suffering from chronic neck muscle pain has recently been documented (Andersen *et al*., 2008b, 2012). However, that study only included women with a clinical diagnosis of trapezius myalgia and therefore the potential beneficial effect of resistance training in a broader group of adults with neck/shoulder muscle pain is relevant to investigate. Furthermore, many adults lack time and motivation to do long-term strenuous sessions of resistance training and the effectiveness of intervention with minimal amount of resistance training remains to be determined. The American College of Sports Medicine (ACSM) (2009) has previously reported that both single- and multiset resistance training approaches can lead to maximal strength gains in the healthy individual but to our knowledge rapid force development has not been studied in populations suffering from musculoskeletal pain.

The aim of our study was to determine the effect of small daily amounts of progressive resistance training on rapid force development of painful neck/shoulder muscles.

## Methods

### Study design

We performed a randomized controlled trial in Copenhagen, Denmark from August to December 2009. The study design and the results on pain and peak torques have been reported previously (Andersen *et al*., 2011). This paper presents secondary analysis on rapid force development, and we have included previous results on peak torques for comparison.

In brief, a screening questionnaire went out to 1094 employees from two large office companies, and a total of 653 (60%) responded to the questionnaire. The target group for this study was full-time office workers with soft tissue tenderness of the neck/shoulder. To avoid competing diseases, exclusion criteria were a medical history of cardiovascular or cerebrovascular events, fibromyalgia, rheumatoid arthritis, cervical disc herniation, whiplash or other significant traumatic injuries of the neck or shoulder, major chronic diseases, pregnancy, working less than 30 hours per week or performing more than 2 hours per week of vigorous physical exercise.

Following initial exclusion criteria, screening employees with self-reported neck/shoulder pain intensity of at least 2 on a scale of 0–10 during the last 3 months, at least 30 day with pain during the last year and self-rated tenderness of the neck/shoulder muscles were invited for a clinical neck/shoulder examination (*n* = 305) (47% of those who replied to the questionnaire) performed by a physical therapist. During the clinical examination, additional exclusion criteria were blood pressure above 160/100, a positive foramen compression test, subacromial impingement syndrome or severe joint pain of the shoulder, elbow or wrist during resisted shoulder abduction. We included employees with a history of frequent neck/shoulder pain during the last year and examiner-verified palpable tenderness in at least one of the examined neck/shoulder muscles (Juul-Kristensen *et al*., 2006) in the trial (*n* = 198) (65% of those who were invited for the examination).

### Participants

All participants (mean age 43·1 (SD: 10·5) years; 174 women and 24 men) were informed about the main objective and content of the project and gave written informed consent to participate in the study. The study was approved by the Local Ethical Committee (HC2008103) and was registered in the International Standard Randomized Controlled Trial Number Register: ISRCTN60264809. Concealed random allocation to one of the three intervention groups was performed after clinical examination of all participants as previously described (Andersen *et al*., 2011). Table 1 shows baseline demographics after allocation to the three groups as well as the physiological characteristics of the trapezius muscle.

**Table 1:** Baseline demographics and physiological characteristics of the trapezius muscle after allocation to the three groups. Results are mean (SD)

	TG2 (*n* = 66)	TG12 (*n* = 66)	CON (*n* = 66)
Demographics			
Age (year)	44·3 (10·9)	42·2 (10·8)	42·9 (9·9)
Height (cm)	171·1 (7·8)	169·5 (7·6)	169·2 (7·2)
Weight (kg)	72·4 (13·6)	68·3 (14·4)	66·6 (11·2)
Body Mass Index (kg × m^−2^)	24·7 (4·5)	23·7 (4·7)	23·2 (3·6)
Physiological characteristics of trapezius muscle			
Peak torque (PT; Nm)	44·3 (13·0)	43·8 (13·8)	43·8 (12·9)
Rate of torque development (RTD; Nm × s^−1^)	194·1 (105·5)	182·3 (99·4)	206·0 (124·3)
Muscle pain (VAS; 0–10 scale)	3·5 (1·6)	3·8 (2·1)	3·5 (1·7)

### Test procedure and dynamometry

The blinded examiner determined the participants’ muscle strength as the maximal torque value of five attempts exerted during maximal voluntary shoulder abduction at a static 90-degree shoulder joint angle against a Bofors dynamometer (Bofors Elektronik, Karlskoga, Sweden). The Bofors force transducer was adjusted so the articulating joint line between the radial row and the proximal row of carpal bones was placed in the middle of the dynamometer.

The participants were instructed to press as hard and fast as possible with verbal encouragement for approximately 3 s against the force transducer allowing for 20 s of rest between trials. We sampled the signal at 100 Hz and saved it for offline analysis in Microsoft Excel via LabView 7.1 (National Instruments, TX, USA). We also measured the participants’ lever arm to calculate torque around the shoulder joint: defined as the distance from the contact area of the Bofors dynamometer on the wrist to the acromion of the scapula.

### Outcomes

For each trial, the peak torque (PT; unit Nm) and rate of torque development (RTD; unit Nm s^−1^) were determined as the maximal value of the torque-time and the steepest slope over 100 ms of the rising part of the torque-time curve, that is, determined as the peak value of a moving window of 100 ms (ΔTorque/ΔTime), respectively. The highest obtained values for PT and RTD were selected for statistical analysis.

### Interventions

This study has three arms; 2 min of progressive resistance training performed five times a week, 12 min of progressive resistance training performed five times a week and a control group receiving weekly information on general health. All three interventions were initiated simultaneously and lasted 10 weeks. The intervention activities have been described in detail previously (Andersen *et al*., 2011). In brief, the 2- and 12-min groups performed shoulder abductions in the scapular plane – also known as ‘lateral raise’ – to effectively target several relevant neck/shoulder muscles (Andersen *et al*., 2008b, 2010). Physical therapists taught the participants to perform the training exercise in a controlled manner, raising and lowering the arms in approximately 2 s, respectively. The 12-min group performed 5–6 sets of 8–12 repetitions in a progressive manner – based on general recommendations by the ACSM (2009). Both groups performed the exercise repetitions at a slow and controlled pace taking approximately 2 s for the concentric phase, 0 s at the apex of the lift and 2 s for the eccentric phase as it is commonly instructed in the fitness industry. The 2-min group was instructed from day one to reach complete failure during training and the participants were encouraged to try and beat their previous best each training session. The training load applied corresponded to 8–12 RM and the load was increased – by the introduction of a thicker elastic tubing – once the participants could perform the 12 repetitions with relative ease. After an introductory week, the training was unsupervised for the remainder of the intervention period and the participants logged all training in a diary. The control group received e-mail based information once a week during the 10-week intervention period on various aspects of general health (e.g. diet, smoking, alcohol, physical exercise, stress management, workplace ergonomics and indoor climate).

### Statistics

Variables were analysed in accordance with the CONSORT statement for randomized controlled trials intention-to-treat principle. Dropouts were invited to participate in the follow-up test to avoid selection bias. Between-group differences were determined by analysis of variance using the mixed procedure of SAS (SAS institute, Cary, NC, version 9.2). Pearson’s correlation was used to analyse relationships between changes in RTD and PT to pain.

An alpha level of 5% was accepted as statistically significant. We report baseline results as means (SD) and changes from baseline to follow-up as means (95% confidence intervals) unless otherwise stated.

## Results

Table 1 shows that at baseline the participants in the three groups were comparable regarding demographic and clinical characteristics. Adherence was satisfactorily high; participants exercised on average more than three times per week. Dropouts were 5-, 6- and 2- participants from the 2 min, 10 min and control group, respectively, thus leaving 185 participants to be included in the analysis. Inter Class Coefficient (ICC) between first and second test round was 0·89 for RTD. The ICC for PT has previously been reported (Andersen *et al*., 2011).

### Rate of torque development

Compared to the control group, RTD increased 31·0 Nm s^−1^ from 194·1 (105·5) Nm s^−1^ to 225·1 (119·5) Nm s^−1^ in the 2-min group [95% confidence interval: (1·33–11·80)] and 33·2 from 182·3 (99·4) Nm s^−1^ to 215·4 (121·8) Nm s^−1^ in the 12-min group [95% confidence interval: (1·66–12·33)], corresponding to an increase of 16·0% and 18·2% in the two groups, respectively ( Table 2). In the control group, RTD decreased 1·8%, which was non-significant (*P* = 0·6). A significant but weak relationship existed between changes in rapid force development and self-reported pain pre- to postintervention (*r* = 0·27, *P*<0·01).

**Table 2:** Difference from baseline to 10-week follow-up for rate of torque development (Nm × s^−1^) and peak torque (Nm). Results are mean (95% confidence interval). 95% confidence intervals for between-group differences of TG2 to control and TG12 to control are also shown

	Difference from baseline to 10-week follow-up			Between-group differences	
Outcome measure	TG2	TG12	Control	TG2 to control mean (95% CI)	TG12 to control mean (95% CI)
Muscle testing					
Rate of torque development (Nm × s^−1^)	31·0 (3·08–10·62)	33·2 (3·38–11·17)	−3·7 (−3·38–3·94)	34·7 (1·33–11·80)	36·9 (1·66–12·33)
Peak torque (Nm)	2·5 (0·40–0·89)	2·2 (0·35–0·86)	0·5 (0·02–0·49)	2·0 (0·05–0·73)	1·8 (0·006-0·70)

### Peak torque

Compared with the control group maximal muscle strength as measured by PT increased 2·5 Nm from 44·3 (13·0) Nm to 46·8 (14·4) Nm in the 2-min group [95% confidence interval: (0·05–0·73)] and 2·2 Nm from 43·8 (13·8) Nm to 46·0 (14·3) Nm in the 12-min group [95% confidence interval: (0·01–0·70)], corresponding to an increase of 5·7% and 5·1% in the two groups, respectively (Table 2). The control group had a non-significant increase in PT of 1·2% corresponding to 0·5 (3·7) Nm from 43·8 (12·9) Nm to 44·3 (13·6) Nm. No significant relationship existed between changes in maximal muscle strength and self-reported pain.

## Discussion

Our study shows significant increases in RTD following two different brief high frequency training protocols in people with neck/shoulder pain compared to controls receiving information on general health. Changes in maximal muscle strength did also increase, although to a less extent (5–6%). Furthermore a correlation analysis showed a significant relationship between changes in RTD, but not PT, and self-reported pain.

Previous studies have shown a positive effect on neck/shoulder strength following typical resistance training protocols (Ylinen *et al*., 2003; Chiu *et al*., 2005; Andersen *et al*., 2008b) and Ylinen & Ruuska, (1994) have previously reported a positive relationship between increases in maximal strength and reductions in musculoskeletal pain. It is generally recognized that significant muscle strength increases can be obtained quite fast, and prolonged exposure to resistance training improves neural drive (Narici *et al*., 1989; Häkkinen *et al*., 1998; Aagaard *et al*., 2000) and firing frequency (Cutsem *et al*., 1998) along with a host of structural changes including: increased anatomical cross-sectional area (CSA) (Higbie *et al*., 1996; Aagaard *et al*., 2002; Reeves & Maganaris, 2003), increased physiological CSA of individual muscle fibres and increased number of myosin heavy chain type IIa filaments (Staron *et al*., 1994) as well as architectural changes of the muscle (Reeves & Maganaris, 2003; Suetta *et al*., 2004). To obtain changes in neural function following resistance training of pain-free muscles, the typical recommendations include loading parameters around 65% 1RM for untrained individuals (ACSM 2009) as well as the intention of achieving the highest possible movement velocity during each repetition (Behm & Sale, 1993). By contrast, the instruction in our study was to perform the exercise in a controlled manner, avoiding jerkiness and sudden acceleration. Thus, RTD may not have increased due to typical adaptations seen in pain-free individuals, but rather due to disinhibition of pain on the ability to rapidly activate the muscles. Although a positive association between changes in pain and RTD partially confirmed this, it should also be noted that the correlation was relatively weak. Our research team has previously reported the primary outcomes of this investigation (Andersen *et al*., 2011) where significant and clinically relevant reductions in neck/shoulder pain were observed following the 2-min- and 12-min training protocols. Together with the results of the present study showing significant improvements in RTD of roughly 16–18% across intervention groups confirms the theory of the impact positive changes in pain has on muscle function.

In individuals not experiencing musculoskeletal pain the physiological mechanisms controlling RTD include: neural drive to the muscles (Narici *et al*., 1989; Häkkinen *et al*., 1998; Aagaard *et al*., 2000), amount of motor neuron inhibition, rate of EMG rise (Aagaard *et al*., 2002; Del & Cafarelli, 2007), motor neuron excitability (Aagaard *et al*., 2002; Del & Cafarelli, 2007), firing frequency and the presence of double spikes (Cutsem *et al*., 1998) but for people experiencing musculoskeletal pain and discomfort, rapid force development may be limited by the fear of pain (Al-Obaidi *et al*., 2000; Reneman *et al*., 2007). Pain is a sensory and emotional experience associated with actual or potential tissue damage and is produced by the output of a widely distributed neural network in the brain (Melzack, 1999). After investigating painful and adjacent upper extremity muscles, Andersen *et al*., (2008c) suggested that a *feedforward* mechanism limits neural drive to painful muscles during rapid contractions as well as to its synergists, and thereby limits RTD, due to a ‘fear of pain’ output from the brain. Complementing this function, Andersen *et al*., (2008c) also suggests that a *feedback* mechanism is responsible for the impairment of painful muscles during maximal force development coming from muscle spindles and Golgi tendon organs (Moore, 1984). Given the extensive impact ‘the pain experience’ can have on body functioning (Swinkels-Meewisse *et al*., 2006), it is not unlikely that people with musculoskeletal pain and discomfort have decreased rapid force development as a direct consequence of the painful event, and by decreasing pain, RTD will increase as we have observed in the present study. This suggests that a modulation of fear during resistance training is taking place. It has been suggested that specific neural networks in the brain act as threat management systems governing actual or perceived threats to the structural integrity of the tissue of the body (Visser & Davies, 2010). The neural network can generate protective sensorimotor responses such as limb weakness or pain (Visser & Davies, 2010). In the present study, the training intervention of 2- or 12 min of daily resistance training using elastic tubing for either one set with as many consecutive repetitions as possible or 5–6 sets of 8–12 repetitions performed slow and controlled does not conform to the general consensus of heavy and rapid lifting required to induce maximal or explosive strength gains. However, the training frequency in both groups was, compared to the literature, quite high. Ratamess *et al*., (2008) have shown that exposure to high frequency resistance training (4 times per week) is superior to lower frequency strategies (3, 2 and 1 weekly training sessions) when maximal strength capacity is the goal. Similarly, Rhea *et al*., (2002) found that healthy, untrained individuals benefitted greatly from 3 weekly sessions of resistance training. Compared to the studies of Ratamess *et al*., (2008) and Rhea *et al*., (2002), the frequency exposure in our study can be considered very high and may be an important factor when providing possible explanations to the observed increase in RTD particularly and the associated decrease in self-perceived pain. Melzack and Wall’s gate control theory of pain from 1965 suggested that a modulation of inputs in the dorsal horns of the spinal cord could together with other central adaptive mechanisms contribute to reductions in perceived pain (Melzack, 1999). It is commonly known that articulate and muscular tissue contain mechanoreceptors which send proprioceptive information about joint position, speed of movement and load to the brain (Zimny, 1988). During movement these mechanoreceptors are stimulated and could potentially excite inhibitory interneurons, thereby blocking the nociceptive signal and closing the pain gate over time with frequent stimulus. Ultimately, this could lead to less fear of movement, which would cause disinhibition of motor outflow to the working muscles.

Another possible pain reduction mechanism as mentioned by Jay *et al*. (2011) in a study investigating the effects of explosive resistance training on non-chronic muscle pain is the desensitization of chemo nociceptive nerve endings by local functional hyperaemia mechanisms normalizing intramuscular metabolite concentrations. Flushing of metabolite build-up might also play a roll locally in the muscles when looking for possible explanations to reductions in self-reported pain following light and frequent resistance training as in the present study. Based on the above, it seems plausible that both central modulatory effects and local muscular events and adaptations could contribute to the altered pain experience and fear of rapid contractions thereby at least partially explain the significant increases in rapid force development we observed in this study.

Finally, it is interesting that there were no differences in RTD or PT between the two training groups. Obviously this makes the training protocol of the 2-min group the most time efficient protocol to choose especially if we put physical training and rehabilitation into an implementation context in the working environment. Furthermore, the lack of difference in RTD and PT between the two training groups may suggest that training frequency is more important than training volume for the untrained individual in a rehabilitation setting.

Our study has both strengths and limitations. A significant strength of the present study is the high ICC between first and second test round of both RTD and PT. A limitation is that we did not measure fear of movement or fear avoidance behaviour in relation to rapid arm movement. This could have been done either by asking the participants just before initiating the contraction during pre- and posttesting, if they are expecting the movement to be painful or monitoring and recording galvanic skin response, heart rate, blood pressure, pupil dilation and skin tonus and colour.

## Conclusion and practical implication

We have shown that rapid force development is a more sensitive measure than maximal force capacity when investigating musculoskeletal pain of the upper extremity. The use of rapid force development tracking in clinical rehabilitation settings may be a more valuable assessment as quick movements, which occur in sports and normal everyday life both, require a rapid force capacity and is often associated with a cortical ‘fear of pain’ output, thus creating neural inhibition to the engaging muscles. Additionally, we have also shown that a very brief (one set), but frequent (5 day per week), training stimulus have a positive effect on rapid force development in people with musculoskeletal pain.

## Conflict of interest

The authors have no conflicts of interest.
